# Association between objectively assessed sedentary time and physical activity with metabolic risk factors among people with recently diagnosed type 2 diabetes

**DOI:** 10.1007/s00125-013-3069-8

**Published:** 2013-10-03

**Authors:** Andrew J. M. Cooper, Soren Brage, Ulf Ekelund, Nicholas J. Wareham, Simon J. Griffin, Rebecca K. Simmons

**Affiliations:** 1 MRC Epidemiology Unit, University of Cambridge, Institute of Metabolic Science, Box 285, Addenbrooke’s Hospital, Hills Road, Cambridge, CB2 0QQ UK; 2 Department of Sport Medicine, Norwegian School of Sport Sciences, Ullevål Stadion, Oslo, Norway; 3 Primary Care Unit, Institute of Public Health, University of Cambridge, Cambridge, UK

**Keywords:** ADDITION-Plus, Cardiovascular risk, Cohort, Physical activity, Sedentary, Type 2 diabetes

## Abstract

**Aims/hypothesis:**

The aim of our study was to examine the associations between sedentary time (SED-time), time spent in moderate-to-vigorous-intensity physical activity (MVPA), total physical activity energy expenditure (PAEE) and cardiorespiratory fitness with metabolic risk among individuals with recently diagnosed type 2 diabetes.

**Methods:**

Individuals participating in the Anglo-Danish-Dutch Study of Intensive Treatment in People with Screen Detected Diabetes in Primary Care (ADDITION)-Plus trial underwent measurement of SED-time, MVPA and PAEE using a combined activity and movement sensor (*n* = 394), and evaluation of cardiorespiratory fitness (*n* = 291) and anthropometric and metabolic status. Clustered metabolic risk was calculated by summing standardised values for waist circumference, triacylglycerol, HbA_1c_, systolic blood pressure and the inverse of HDL-cholesterol. Multivariate linear regression analyses were used to quantify the associations between SED-time, MVPA, PAEE and cardiorespiratory fitness with individual metabolic risk factors and clustered metabolic risk.

**Results:**

Each additional 1 h of SED-time was positively associated with clustered metabolic risk, independently of sleep duration and MVPA (β = 0.16 [95% CI 0.03, 0.29]). After accounting for SED-time, MVPA was associated with systolic blood pressure (β = −2.07 [−4.03, −0.11]) but not with clustered metabolic risk (β = 0.01 [−0.28, 0.30]). PAEE and cardiorespiratory fitness were significantly and independently inversely associated with clustered metabolic risk (β = −0.03 [−0.05, −0.02] and β = −0.06 [−0.10, −0.03], respectively). Associations between SED-time and metabolic risk were generally stronger in the low compared with the high fitness group.

**Conclusions/interpretation:**

PAEE was inversely associated with metabolic risk, whereas SED-time was positively associated with metabolic risk. MVPA was not associated with clustered metabolic risk after accounting for SED-time. Encouraging this high-risk group to decrease SED-time, particularly those with low cardiorespiratory fitness, and increase their overall physical activity may have beneficial effects on disease progression and reduction of cardiovascular risk.

*Trial registration*: ISRCTN99175498

**Electronic supplementary material:**

The online version of this article (doi:10.1007/s00125-013-3069-8) contains peer-reviewed but unedited supplementary material, which is available to authorised users.

## Introduction

Physical inactivity is an important modifiable lifestyle risk factor associated with hyperglycaemia, hypertension, dyslipidaemia and the risk of developing cardiovascular disease (CVD) in both healthy individuals and those with type 2 diabetes [1–5]. Despite the clear health benefits of physical activity (PA), however, individuals with type 2 diabetes generally have low levels of PA [6, 7] and have difficulty maintaining the required level necessary for improving metabolic health [8]. Emerging evidence suggests that targeting sedentary time (SED-time) may represent an additional approach to reducing metabolic risk in apparently healthy populations [9–11]. The assessment of PA and SED-time in these studies, however, has typically been derived from self-report measures. The imprecision and bias associated with self-report measures means that their ability to accurately assess total activity, as well as PA intensity and SED-time, is limited. The single study using an objective measure of PA in individuals with type 2 diabetes showed that SED-time was positively associated with metabolic risk, whereas moderate-to-vigorous-intensity physical activity (MVPA) was only weakly inversely associated with risk [12]. Given the limited amount of time spent in MVPA in this population, and the large amount of SED-time, targeting reductions in SED-time by increasing overall physical activity energy expenditure (PAEE), independently of MVPA, may offer a more feasible strategy to managing metabolic risk. Before advocating trials aimed at reducing SED-time in this population, however, it is important first to elucidate whether this association is independent of sleep duration, which might confound the association with metabolic risk [13]. As the association between different dimensions of PA and metabolic risk may differ by level of cardiorespiratory fitness [14], it is also important to establish whether these associations are modified by cardiorespiratory fitness.

Using data from the ADDITION-Plus trial of individuals with type 2 diabetes, which included objective measures of PA using a combined heart rate and movement sensor, we aimed to: (1) examine the magnitude, direction and relative associations between SED-time, MVPA, PAEE and cardiorespiratory fitness with metabolic risk; and (2) determine whether associations of SED-time, MVPA and PAEE differ by level of cardiorespiratory fitness.

## Methods

The design and rationale for the ADDITION-Plus study have been reported previously [15]. In brief, ADDITION-Plus is a randomised controlled trial nested within the intensive treatment arm of the ADDITION-Cambridge study, which evaluated the efficacy of a facilitator-led, theory-based behaviour change intervention for recently diagnosed type 2 diabetic patients. Thirty-four general practices in East Anglia participated in the study. Eligible individuals were those aged 40–69 years who had been diagnosed with type 2 diabetes following screening in the ADDITION study or clinically diagnosed during the previous 3 years in participating general practice surgeries. Exclusion criteria were women who were pregnant or lactating or those with a likely survival prognosis of less than 1 year. Out of 1,109 eligible individuals, 478 agreed to participate in ADDITION-Plus and were individually randomised to receive either intensive treatment alone (*n* = 239) or intensive treatment plus the facilitator-led individual behaviour change intervention (*n* = 239). The two trial arms were pooled and a cohort analysis conducted. All participants gave written informed consent, and the study was approved by the Eastern Multi-Centre Research Ethics Committee (reference number 02/5/54). The trial is registered as ISRCTN99175498.

### Assessment of PA, cardiorespiratory fitness, sleep duration and metabolic risk factors

Assessment of ADDITION-Plus participants included physiological and anthropometric measures by trained staff following standard operating procedures, venesection and completion of self-report questionnaires. PA was assessed using a combined heart rate and movement sensor (Actiheart; CamNtech, Cambridge, UK) worn continuously for 4 days in 30 s resolution [16]. A graded treadmill walk test was used to calibrate heart rate individually [17] and to estimate cardiorespiratory fitness by extrapolation of the heart rate/oxygen consumption relationship to age-predicted maximal heart rate. For participants who did not complete an individual calibration test (*n* = 170), we used all valid calibration tests in the rest of the sample (*n* = 308) to derive a group calibration equation adjusted for age, sex, beta blocker (yes = 94, no = 214) and sleeping heart rate for the translation of heart rate into activity intensity. Heart rate data collected during the free-living period were processed using noise classification followed by Gaussian robust regression [18], and average activity intensity (J min^−1^ kg^−1^) was estimated using a branched equation framework [19]. Resulting time-series data were summarised into PAEE (kJ kg^−1^ day^−1^), SED-time and MVPA (min/day), whilst minimising diurnal information bias caused by non-wear periods (segments of non-physiological data). Cardiorespiratory fitness ($\dot{V}{\mathrm{O}}_{2 \max .\mathrm{pred}}$) was assessed for 291 participants (196 men and 95 women) using extrapolation of heart rate response to a submaximal ramped exercise test to age-predicted maximum heart rate (208–0.7 × age), as described elsewhere [17]. SED-time was defined as a metabolic equivalent of task value (MET value) of <1.5, in accordance with current convention [20], and MVPA as ≥3.0 MET, both primarily using the Oxford estimate of resting metabolic rate (RMR) to define 1 MET [21], and secondarily using a fixed value of 20.35 J ml O_2_ × 3.5 ml O_2_ min^−1^ kg^−1^. Sensitivity analyses were run to test the robustness of results with regard to MET thresholds (<1.75 MET vs <1.5 MET for SED-time, and ≥4.0 MET vs ≥3.0 MET for MVPA). We overlaid self-reported sleep timings on the objective time-series data, the appropriateness of which was verified by visual inspection (see Methods, Covariate assessment, for a full explanation). From these data, we calculated sleep duration and non-sleep SED-time.

Blood pressure was calculated as the mean of three measurements performed after 10 min of rest and with participants seated with a cuff placed on the predominant arm at the level of the heart, using an automatic sphygmomanometer (Omron M4; Milton Keynes, UK). Body weight and height were measured in light clothing and without shoes using a scale (SECA; Birmingham, UK) and a fixed rigid stadiometer, respectively. Waist circumference was calculated as the average of two measurements taken halfway between the lowest point of the rib cage and the anterior superior iliac crest while standing. HbA_1c_ was measured in venous samples using an ion-exchange high-performance liquid chromatography method (Tosoh Bioscience, Redditch, UK). Serum total cholesterol, HDL-cholesterol and triacylglycerol were measured using enzymatic techniques (Dade Behring Dimension Analyzer; Dade Behring, Newark, NJ, USA). Standardised self-report questionnaires were used to collect information on sociodemographic characteristics and sleep duration.

### Covariate assessment

Medication adherence was assessed by the Medication Adherence Report Schedule (MARS) questionnaire [22]. Smoking status was assessed by questionnaire with a yes/no answer to being a: current smoker, former smoker or never smoker. Dietary intake was evaluated using a validated food frequency questionnaire [23]. Occupational socioeconomic class was evaluated by questionnaire and was subsequently collapsed into three classes: (1) higher managerial, administrative and professional occupations; (2) intermediate occupations; and (3) routine and manual occupations [24]. Habitual bedtime and wake time were self-reported for weekdays and weekends using the EPIC–Norfolk Physical Activity Questionnaire (EPAQ2) [25]. Sleep duration was calculated as (5/7 × weekday sleep duration) + (2/7 × weekend sleep duration). We overlaid the self-reported sleep timings on the time-series data from the combined heart rate and movement sensor to visually identify participants who had high motion/heart rate periods (which we identified as being indistinguishable from time spent awake and in contrast to combined heart rate and movement sensor data during time spent sleeping) exceeding their self-reported bedtimes and/or wake times by ≥30 min. Agreement between the self-reported bedtimes and wake times with the objective time-series data was assessed by two examiners independently for each participant (A. J. M. Cooper and K. Westgate [Medical Research Council Epidemiology Unit, Cambridge, UK]). The kappa statistic for agreement between examiners was 0.77. Disagreement between examiners was resolved by re-examination of the data and discussion. In total, self-reported bedtimes and wake times were deemed to be incompatible with the objective time-series data for 21 participants, who were excluded from analyses.

### Calculation of the clustered metabolic risk z score

A summary score of clustered metabolic risk (zMS) was calculated by summing standardised values for waist circumference, fasting triacylglycerol, HbA_1c_ and systolic blood pressure and the inverse of HDL-cholesterol. Variables were standardised by subtracting the sample mean from the individual mean and dividing by the SD.

Complete data on objectively measured PA, metabolic risk factors and potential confounding variables were available for 394 participants (*n* = 291 for cardiorespiratory fitness).

### Statistical analysis

Descriptive characteristics were summarised separately for men and women using means with SDs, medians with interquartile ranges (IQR) or frequencies. Wilcoxon rank-sum tests, *t* tests or *χ* ^2^ tests were used to examine whether there were any differences in participant characteristics between those with and those without missing data. Fasting triacylglycerol values were log transformed (base *e*) due to their non-normal distribution. Associations between SED-time, MVPA, PAEE, $\dot{V}{\mathrm{O}}_{2 \max .\mathrm{pred}}$ and sleep duration were estimated using Pearson correlation coefficients.

We used multivariate linear regression analyses to model the associations between SED-time (h/day), MVPA (h/day), PAEE (kJ kg^−1^ day^−1^) and $\dot{V}{\mathrm{O}}_{2 \max .\mathrm{pred}}$ (ml O_2_ kg^−1^ min^−1^) with individual subcomponents of the zMS (waist circumference, fasting triacylglycerol, HbA_1c_, systolic blood pressure and inverted HDL-cholesterol), and with the clustered metabolic risk score as one variable after confirming that our data met the assumptions underlying linear regression (i.e. linearity, normality, homoscedasticity and absence of multicolinearity). To compare directly the relative contribution of SED-time, MVPA, PAEE and $\dot{V}{\mathrm{O}}_{2 \max .\mathrm{pred}}$ we also expressed these exposure variables in the same units (per SD difference). Under the assumption that these variables are measured with the same degree of measurement error, their expression in standardised form allows direct comparisons of the magnitude of association between measures and clustered metabolic risk. All regression models are presented unadjusted (crude) and adjusted for age, sex, intervention group, occupational socioeconomic class, smoking status, sleep duration, total energy intake, percentage of energy from fat, alcohol intake and waist circumference (except when waist circumference or zMS were modelled as the outcome). When the outcome of interest was blood pressure, HbA_1c_, triacylglycerol or HDL-cholesterol, we additionally adjusted for the use of antihypertensive, glucose-lowering or lipid-lowering medication, respectively. For the zMS we adjusted for the use of antihypertensive, glucose-lowering and lipid-lowering medications. Finally, to examine the association between SED-time (independently of MVPA) and MVPA (independently of SED-time) with subcomponents of the zMS and the score as one variable, we additionally adjusted for MVPA and SED-time, respectively.

All data were analysed in continuous form, although some data were categorised into tertiles for illustrative purposes. Finally, we examined whether the associations with SED-time, MVPA, PAEE and $\dot{V}{\mathrm{O}}_{2 \max .\mathrm{pred}}$ with zMS were modified by age (<60 vs ≥60 years) and sex. We also examined whether the associations of SED-time, MVPA and PAEE with metabolic risk factors and clustered metabolic risk were modified by cardiorespiratory fitness levels (continuous). In further sensitivity analyses we examined whether our results would have differed by excluding participants with fewer than 3 days of combined heart rate and movement sensor data (*n* = 21), or by excluding participants who did not complete an individual calibration test (*n* = 103).

All statistical analyses were performed using Stata/SE 12.1 (StataCorp LP, College Station, TX, USA).

## Results

Table 1 shows the anthropometric and metabolic characteristics of participants with complete data at 1 year (*n* = 394), stratified by sex. The mean (SD) age of men and women was 60.2 (7.4) and 60.5 (7.4) years, respectively. More men than women met the inclusion criteria and agreed to participate in the study. Participants with missing data (*n* = 84) had similar baseline values for all anthropometric and metabolic characteristics shown in Table 1 (data not shown). As expected, men were taller and heavier than women, had a larger waist circumference and reported consuming a greater amount of alcohol. Men had higher systolic blood pressure and triacylglycerol levels compared with women, whereas women had higher total and HDL-cholesterol levels. Total PAEE was higher among men than among women (37.6 vs 29.6 kJ kg^−1^ day^−1^, respectively; *p* < 0.001) and men had higher levels of cardiorespiratory fitness than women (33.4 ml O_2_ kg^−1^ min^−1^ vs 25.2 ml O_2_ kg^−1^ min^−1^, respectively; *p* < 0.001). Men and women had similar amounts of SED-time (9.1 vs 9.0 h/day, respectively; *p* = 0.69), but men reported spending fewer hours sleeping (8.1 vs 8.7 h/day, respectively; *p* < 0.001) and were more likely than women to spend time in MVPA (*p* = 0.007). SED-time was strongly inversely correlated with time spent in MVPA (*r* = −0.60; *p* < 0.001) and with PAEE (*r* = −0.76; *p* < 0.001). SED-time was weakly inversely correlated with sleep duration (*r* = −0.28; *p* < 0.001). Cardiorespiratory fitness was moderately correlated with MVPA (*r* = 0.44; *p* < 0.001) and PAEE (*r* = 0.49; *p* < 0.001), and weakly inversely correlated with SED-time (*r* = −0.11; *p* < 0.001). MVPA was strongly correlated with PAEE (*r* = 0.90; *p* < 0.001).

**Table 1:** Anthropometric and metabolic characteristics of ADDITION-Plus participants (*n* = 394), stratified by sex

Characteristic	Men (*n* = 250)	Women (*n* = 144)	*p* value for difference between groups
Age, years	60.2 (7.4)	60.5 (7.4)	0.71
Occupational socioeconomic class, %			0.01
Managerial	47.2	31.9	
Intermediate	22.0	30.6	
Routine and manual	30.8	37.5	
Height, cm	174.9 (7.2)	161.4 (6.9)	<0.001
Weight, kg	96.9 (16.7)	85.9 (17.7)	<0.001
BMI, kg/m^2^	31.6 (5.1)	32.9 (6.0)	0.03
Waist circumference, cm	111.3 (12.5)	105.0 (13.0)	<0.001
Systolic blood pressure, mmHg	132.0 (16.7)	125.9 (17.0)	<0.001
Triacylglycerol, mmol/l^a^	1.7 (1.2–2.3)	1.6 (1.1–2.1)	0.06
Total cholesterol, mmol/l	4.2 (0.9)	4.4 (0.8)	0.008
HDL-cholesterol, mmol/l	1.1 (0.3)	1.3 (0.3)	<0.001
HbA_1c,_ %	6.7 (1.0)	6.6 (0.8)	0.68
HbA_1c,_ mmol/mol	49.7	48.6	
On glucose-lowering medication, %	49.6	54.9	0.31
On antihypertensive medication, %	72.4	70.1	0.63
On lipid-lowering medication, %	74.0	79.9	0.19
Total energy intake, kJ/day^a^	7,272 (6,130, 8,489)	6,602 (5,711, 8,489)	0.06
Percentage of energy from fat, %	30.6 (5.8)	30.9 (5.7)	0.62
Alcohol intake, g/day^a^	6.0 (1.0, 15.0)	2.0 (0.0, 5.0)	<0.001
PAEE, kJ kg^-1^ day^-1 b^	37.6 (18.2)	29.6 (13.5)	<0.001
Average sleep duration, h/day	8.1 (1.1)	8.7 (0.9)	<0.001
Duration SED-time, h/day^b,c^	9.1 (2.7)	9.0 (2.4)	0.69
Duration SED-time, h/day^b,d^	10.3 (2.6)	10.5 (2.2)	0.50
Duration MVPA, h/day^b,c^	1.4 (1.2)	1.1 (0.9)	0.007
Duration MVPA, h/day^b,d^	0.88 (0.92)	0.43 (0.48)	<0.001
$\dot{V}{\mathrm{O}}_{2 \max .\mathrm{pred}}$, ml O_2_ kg^−1^ min^−1a,e^	33.4 (28.1, 37.4)	25.2 (20.2, 31.9)	<0.001

Data are means (SD) unless stated otherwise

^a^Median (IQR)

^b^Individually calibrated where available (*n* = 291), otherwise group calibrated (*n* = 103)

^c^1 MET defined using individual estimate of RMR [21]

^d^1 MET defined using 71.2 J min^−1^ kg^−1^

^e^ $\dot{V}{\mathrm{O}}_{2 \max .\mathrm{pred}}$ available for 291 participants (196 men and 95 women)

**Fig. 1:**
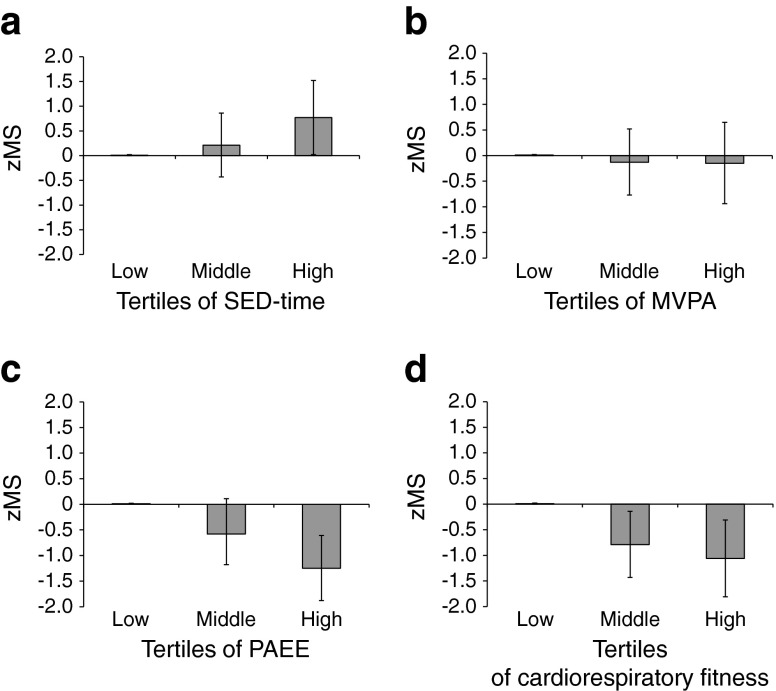
Associations of SED-time, MVPA, PAEE and cardiorespiratory fitness with clustered metabolic risk. Mean SED-time in the lower (reference), middle and higher tertile groups was 6.4 (range 1.2–8.0), 9.1 (range 8.0–10.4) and 11.6 (range 10.4–16.4) h/day, respectively (**a**). Mean time spent in MVPA in the lower, middle and higher tertile groups was 0.4 (range 0.0–0.6), 1.0 (range 0.6–1.5) and 2.4 (range 1.5–6.1) h/day, respectively (**b**). Mean PAEE in the lower, middle and higher tertile groups was 18.9 (range 5.6–24.7), 31.2 (range 24.8–39.4) and 50.3 (range 39.6–98.8) kJ kg^−1^ day^−1^, respectively (**c**). Mean cardiorespiratory fitness in the lower (reference), middle and higher tertile groups was 21.0 (range 11.3–26.9), 31.0 (range 27.1–34.6) and 38.7 (range 34.8–58.6) ml O_2_ kg^−1^ min^−1^, respectively (**d**). Data are the zMS for each group (95% CI), adjusted for age, sex, intervention group, occupational socioeconomic class, smoking status, sleep duration, total energy intake, percentage energy from fat, alcohol intake, and use of antihypertensive drugs, glucose-lowering drugs and lipid-lowering drugs. SED-time is additionally adjusted for MVPA, and MVPA is additionally adjusted for SED-time. *p* values for trend across categories for SED-time (**a**), MVPA (**b**), PAEE (**c**) and cardiorespiratory fitness (**d**) were *p* = 0.04, *p* = 0.72, *p* < 0.001 and *p* = 0.001, respectively

Table 2 shows crude and adjusted associations between SED-time (h/day), MVPA (h/day), PAEE (kJ kg^−1^ day^−1^) and $\dot{V}{\mathrm{O}}_{2 \max .\mathrm{pred}}$ with subcomponents of the zMS and the clustered metabolic risk score as one variable. In adjusted analyses, PAEE was inversely associated with waist circumference (β = −0.27 cm; 95% CI −0.35, −0.20) and clustered metabolic risk (β = −0.03; 95% CI −0.05, −0.02). Results were similar for $\dot{V}{\mathrm{O}}_{2 \max .\mathrm{pred}}$ (Table 2). By contrast, SED-time was positively associated with waist circumference (β = 0.68 cm; 95% CI 0.01, 1.35), triacylglycerol levels (β = 0.03; 95% CI 0.00, 0.06) and clustered metabolic risk (β = 0.16; 95% CI 0.03, 0.29), and inversely associated with HDL-cholesterol levels (β = −0.02; 95% CI −0.03, −0.001) in analyses adjusted for time spent in MVPA. After accounting for SED-time, time spent in MVPA only remained significantly inversely associated with systolic blood pressure (β = −2.07; 95% CI −4.03, −0.11). The bottom row of Table 2 shows crude and adjusted associations between standardised measures (SDs) of PAEE, SED-time and MVPA with clustered metabolic risk. The magnitude of the association between PAEE and metabolic risk was similar to the magnitude between SED-time and metabolic risk, although in the opposite direction, such that each SD increase in PAEE was associated with a lower metabolic risk (β = −0.55; 95% CI −0.81, −0.28), whereas each SD increase in SED-time was associated with a higher metabolic risk (β = 0.42; 95% CI 0.09, 0.75). Figure 1 highlights the association of SED-time, MVPA, PAEE and $\dot{V}{\mathrm{O}}_{2 \max .\mathrm{pred}}$ with metabolic risk after adjustment for potential confounding factors. Table 1 in the electronic supplementary material (ESM) shows the associations between metabolic risk variables with SED-time and MVPA using a fixed value of RMR to define 1 MET; these results were largely similar to those reported using individualised RMR, except for a stronger inverse association between MVPA and waist circumference, which remained significant after adjustment for SED-time.

**Table 2:** Crude and adjusted linear associations between SED-time and time spent in MVPA, PAEE and $\dot{V}{\mathrm{O}}_{2 \max .\mathrm{pred}}$ with subcomponents of metabolic risk and clustered metabolic risk in the ADDITION-Plus trial cohort (*n* = 394)

Metabolic risk	SED-time (h/day), β (95% CI)			MVPA (h/day), β (95% CI)			PAEE (kJ kg^−1^ day^−1^), β (95% CI)		$\dot{V}{\mathrm{O}}_{2 \max .\mathrm{pred}}$ (ml O_2_ kg^−1^ min^−1^), β (95% CI)^a^	
	Crude	Adjusted^b^	Adjusted^b^ + MVPA	Crude	Adjusted^b^	Adjusted^b^ + SED-time	Crude	Adjusted^b^	Crude	Adjusted^b^
Waist, cm	0.80 (0.31, 1.29)*	0.97 (0.46, 1.48)*	0.68 (0.01, 1.35)*	−1.09 (−2.24, 0.07)	−2.01 (−3.18, −0.85)*	−1.03 (−2.54, 0.49)	−0.16 (−0.24, −0.09)*	−0.27 (−0.35, −0.20)*	−0.39 (−0.54, −0.24)*	−0.62 (−0.78, −0.46)*
Systolic blood pressure, mmHg	0.38 (−0.27, 1.03)	0.07 (−0.62, 0.75)	−0.51 (−1.37, 0.36)	−1.86 (−3.36, −0.36)*	−1.36 (−2.89, 0.18)	−2.07 (−4.03, −0.11)*	−0.09 (−0.19, 0.01)	−0.07 (−0.18, 0.04)	−0.16 (−0.37, 0.05)	−0.23 (−0.48, 0.02)
HbA_1c_, %	0.00 (−0.03, 0.04)	0.02 (−0.02, 0.05)	0.03 (−0.02, 0.07)	0.01 (−0.07, 0.09)	0.00 (−0.08, 0.08)	0.04 (−0.07, 0.14)	0.00 (−0.01, 0.01)	0.00 (−0.01, 0.01)	0.00 (−0.01, 0.01)	0.00 (−0.02, 0.01)
Log_*e*_ triacylglycerol, mmol/l	0.01 (−0.01, 0.03)	0.02 (−0.01, 0.04)	0.03 (0.00, 0.06)*	0.02 (−0.03, 0.07)	0.01 (−0.04, 0.06)	0.05 (−0.01, 0.11)	0.00 (0.00, 0.00)	0.00 (0.00, 0.00)	0.00 (0.00, 0.01)	0.00 (−0.01, 0.01)
HDL-cholesterol, mmol/l	0.00 (−0.01, 0.01)	−0.01 (−0.02, 0.002)	−0.02 (−0.03, −0.001)*	−0.02 (−0.05, 0.01)	−0.002 (−0.03, 0.02)	−0.02 (−0.06, 0.01)	−0.001 (−0.003, 0.000)	0.001 (−0.001, 0.002)	−0.006 (−0.010, −0.002)^*^	0.00 (−0.01, 0.00)
zMS	0.11 (0.01, 0.21)*	0.16 (0.06, 0.26)*	0.16 (0.03, 0.29)*	−0.07 (−0.30, 0.17)	−0.22 (−0.45, 0.00)	0.01 (−0.28, 0.30)	−0.01 (−0.03, 0.00)	−0.03 (−0.05, −0.02)*	−0.02 (−0.05, 0.02)	−0.06 (−0.10, −0.03)*
zMS^c^	0.28 (0.02, 0.54)*	0.42 (0.16, 0.67)*	0.42 (0.09, 0.75)*	−0.07 (−0.34, 0.19)	−0.25 (−0.50, 0.00)	0.01 (−0.32, 0.33)	−0.21 (−0.47, 0.05)	−0.55 (−0.81, −0.28)*	−0.15 (−0.46, 0.16)	−0.58 (−0.89, −0.27)*

Values for SED-time and MVPA use individualised RMR [21] as definition for 1 MET

^a^ *n* = 291

^b^All coefficients are adjusted for age, sex, intervention group, occupational socioeconomic class, smoking status, sleep duration, total energy intake, percentage of energy from fat, and alcohol intake. All outcomes except zMS and waist circumference are additionally adjusted for waist circumference. Systolic and diastolic blood pressure are additionally adjusted for the use of antihypertensive drugs (yes/no); HbA_1c_ is additionally adjusted for the use of glucose-lowering drugs (yes/no); triacylglycerol is additionally adjusted for the use of lipid-lowering drugs (yes/no); HDL-cholesterol is additionally adjusted for the use of lipid-lowering drugs (yes/no); and zMS is additionally adjusted for the use of antihypertensive drugs (yes/no), glucose-lowering drugs (yes/no), and lipid-lowering drugs (yes/no). zMS is a continuously distributed variable for clustered metabolic risk calculated by summing standardised values for waist circumference, triacylglycerol, HbA_1c_, systolic blood pressure and the inverse of HDL-cholesterol

^c^Difference in zMS per SD difference in SED-time, MVPA, PAEE or $\dot{V}{\mathrm{O}}_{2 \max .\mathrm{pred}}$

^*^ *p* < 0.05

There was no evidence to suggest that the associations between SED-time, MVPA, PAEE and $\dot{V}{\mathrm{O}}_{2 \max .\mathrm{pred}}$ with zMS were modified by either age (all *p* values ≥0.88) or sex (all *p* values ≥0.32). Although the associations of SED-time, MVPA and PAEE with metabolic risk factors and clustered metabolic risk did not differ by level of cardiorespiratory fitness (all *p* values for interaction ≥0.07, except for the association between PAEE and HbA_1c_ [*p* = 0.03]), our results were suggestive of a stronger association between SED-time and subcomponents of metabolic risk among individuals below the median for cardiorespiratory fitness (ESM Table 2). MVPA was strongly inversely associated with systolic blood pressure among the least fit (β = −4.85 mmHg; 95% CI −9.22, −0.48). The estimated β coefficients and 95% CIs were not significantly different between models if we excluded participants with fewer than 3 days of combined heart rate and movement sensor data (*n* = 21) (all *p* values ≥0.36) or we excluded participants who did not complete an individual calibration test (*n* = 103) (all *p* values ≥0.46). Although our results remained robust with regard to changes in MET thresholds for SED-time, a threshold for MVPA of ≥4.0 METs was more strongly inversely associated (numerically larger β coefficient), albeit non-significantly, with waist circumference, systolic blood pressure and clustered metabolic risk compared with an MVPA threshold of ≥3.0 METs (ESM Table 3).

## Discussion

In this cross-sectional study of individuals with recently diagnosed type 2 diabetes, higher levels of SED-time were associated with higher levels of metabolic risk, independently of measured confounders and time spent in MVPA. In contrast, time spent in MVPA was not significantly associated with any metabolic risk factor except for systolic blood pressure and waist circumference after accounting for measured confounders and SED-time. Total PAEE and $\dot{V}{\mathrm{O}}_{2 \max .\mathrm{pred}}$ were inversely associated with waist circumference and a lower metabolic risk profile, independently of measured confounders. Associations between SED-time and measures of metabolic risk were stronger in those with low fitness levels compared with those in the high fitness group, suggesting that higher levels of cardiorespiratory fitness may provide some protection against the deleterious effects of SED-time. Our findings suggest that decreasing the amount of SED-time and increasing overall PA may have beneficial effects on disease progression and cardiovascular risk in patients with type 2 diabetes.

Our study exhibits several strengths, including the use of standardised measures and objective measurement of PA and SED-time. The few previous studies that have objectively assessed SED-time and/or PA have used either heart rate or movement sensing [12, 26–28]. Deriving PA from heart rate measurements alone can lead to imprecision due to the difficulty in distinguishing time spent resting from time spent engaging in low levels of PA that do not result in elevations in heart rate. By contrast, relying on movement sensing alone is limited due to the fact that acceleration of typically only one body segment is recorded, and as such certain activities will not be registered (e.g. cycling). Using a combined heart rate and movement sensor allowed us to discriminate between both rest and low-intensity locomotor activities across biomechanically different activities. This is important in a study population that spends a considerable amount of time sedentary and very little time in MVPA. Our findings are unlikely to be biased as participants with and without missing data were similar. Finally, by using objective time-series data for sleep duration, verified by overlaying self-reported sleep time, we were able to examine all associations independently of sleep, which cannot be considered the same as SED-time in terms of metabolic risk [29, 30].

Some potential limitations of our study merit discussion. First, as this is a cross-sectional study we cannot infer the direction or the causal nature of the associations. Nevertheless, findings from animal models have shown that a lack of local contractile muscle stimulation, such as when sedentary, can adversely affect triacylglycerol, HDL-cholesterol and other metabolic risk factors as a result of suppression of muscle lipoprotein lipase stimulation [31]. Second, despite the transient increase in heart rate when changing posture from sitting to standing [32, 33], it is difficult to accurately distinguish between time spent sitting and time spent standing using the combined heart rate and movement data acquired in this study, and as such it is possible that time spent standing was misclassified as SED-time and vice versa. Any potential misclassification of standing time as SED-time, however, would likely have led to attenuation of the associations with SED-time. Similarly, the questionnaire used to collect data on habitual bedtime and wake time in this study did not include questions related to naps or an estimate of actual sleep duration, which might have resulted in sleep time being misclassified as SED-time and vice versa. Use of a more precise measure of sleep-related variables, such as polysomnography, might reduce misclassification, but these measures are currently not feasible in epidemiological studies. Third, beneficial associations of MVPA with metabolic risk factors might have been missed as a result of the definition of MVPA, as suggested by the stronger, albeit non-significant, inverse associations with waist circumference, systolic blood pressure and clustered metabolic risk when we used a more stringent cut point of ≥4.0 METs, as opposed to ≥3.0 METs. Associations with MVPA were not materially different when we used a fixed intensity threshold of 142.5 J min^−1^ kg^−1^ above rest (3 METs) to define MVPA, rather than an individualised threshold, except for a stronger inverse association with waist circumference, which remained after adjustment for SED-time. Fourthly, cardiorespiratory fitness was not measured directly with a maximal test but was assessed using extrapolation of heart rate response to a submaximal ramped exercise test to age-predicted maximum heart rate. Finally, we cannot exclude the possibility of residual confounding or confounding by unmeasured factors.

Previous cross-sectional and prospective studies have shown beneficial associations between high levels of objectively assessed total PA with HDL-cholesterol and triacylglycerol, insulin and glucose levels in healthy individuals, while longer durations of SED-time have been adversely associated with waist circumference, insulin levels, triacylglycerol levels and overall metabolic risk [26, 28, 34–37]. Further, measures of glucose homeostasis have also been shown to be detrimentally associated with SED-time among individuals with newly diagnosed type 2 diabetes [12]. However, when the association between 6-month change in SED-time and metabolic risk was examined prospectively, no association with glucose homeostasis was observed [12]. Differences between cross-sectional and prospective findings may be explained by the fact that the sample size of existing prospective studies may be inadequate to detect small but biologically important associations related to changes in these behaviours, especially given the short duration of follow-up. Nevertheless, results of studies with objective measures of PAEE and SED-time are consistent with those of prospective studies which have used self-reported data and have shown that PA is a good predictor of CVD morbidity and mortality [38–41] and that SED-time is associated with an increased risk of CVD events [4, 9]. The association between different dimensions of PA and metabolic risk has been shown to differ by cardiorespiratory fitness in healthy adults free from type 2 diabetes [14]. While our findings suggest that reductions in SED-time may be particularly beneficial among the least fit, they highlight the importance of reducing SED-time and increasing total PA for all patients with type 2 diabetes. The present results extend previous observations by using individually calibrated combined heart rate and movement sensing, which has been shown to have high validity for estimating PAEE in both laboratory [42] and free-living conditions [43] and which overcomes many of the limitations associated with either heart rate or movement monitoring alone [16]. Further, we demonstrate that the associations between PAEE and SED-time with metabolic risk are independent of sleep duration.

It is important to consider the clinical implications of the observed association between SED-time and the metabolic risk factors. Each 1 h/day increase in SED-time was associated with a 0.68 cm higher waist circumference, a 0.03 mmol/l higher triacylglycerol level and a 0.02 mmol/l lower HDL-cholesterol level. The difference in SED-time between the 25th and 75th percentiles, however, was almost 4 h/day, which suggests that if the association were causal then it is not unreasonable to suggest that moving from the top to the bottom quartile of SED-time could result in a reduction in waist circumference of over 2.5 cm, a reduction in triacylglycerol levels of 0.12 mmol/l and an increase in HDL-cholesterol by 0.08 mmol/l. In context, changes of this magnitude have each been associated with a 3–5% reduction in CVD risk [44–46].

Our findings support the hypothesis that the biological responses to SED-time likely influence metabolic risk through pathways distinct from those which have been implicated for MVPA [47, 48]. Our data also suggest that while adults with type 2 diabetes may gain additional health benefits from participating in MVPA, reducing SED-time and increasing overall activity, even if it is less than the recommended guidelines [49] it will result in important health benefits. Thus, our findings are consistent with the recommendations of the American Diabetes Association [1], highlighting the importance not only of MVPA but also of increasing overall PA at the expense of SED-time. These findings highlight the need to develop interventions to reduce SED-time that might be incorporated into educational programmes for newly diagnosed patients. This will require better information about determinants of SED-time in this group, but in the meantime simple and cheap approaches such as the provision of pedometers might be recommended [50].

In conclusion, total PAEE, but not MVPA, was associated with improved metabolic risk status in this cohort of recently diagnosed type 2 diabetic patients. Individuals with higher levels of SED-time had higher levels of metabolic risk, and this was particularly noticeable among those with low cardiorespiratory fitness levels. Encouraging this high-risk group to decrease their SED-time by increasing overall PA may have beneficial effects on disease progression and reduction of CVD risk. Future research is needed in order to establish the prospective associations between changes in PAEE, SED-time and time spent in MVPA with metabolic risk, as well as to establish the determinants and maintenance of change in this population.

## Electronic supplementary material

Below is the link to the electronic supplementary material.

ESM Table 1 (PDF 49 kb)

ESM Table 2 (PDF 51 kb)

ESM Table 3 (PDF 95 kb)

## Abbreviations

ADDITION: Anglo-Danish-Dutch Study of Intensive Treatment in People with Screen Detected Diabetes in Primary Care
CVD: Cardiovascular disease
IQR: Interquartile range
MET value: Metabolic equivalent of task value
MVPA: Moderate-to-vigorous-intensity physical activity
PA: Physical activity
PAEE: Physical activity energy expenditure
RMR: Resting metabolic rate
SED-time: Sedentary time
zMS: Standardised clustered metabolic risk score
