# Supplementary material for: Association between objectively assessed sedentary time and physical activity with metabolic risk factors among people with recently diagnosed type 2 diabetes
Source: Diabetologia. 2013 Oct 3;57(1):73–82. doi: 10.1007/s00125-013-3069-8 (PMC3857880; doi:10.1007/s00125-013-3069-8)
Supplement: Supplementary file 3 — (PDF 95 kb) [file 125_2013_3069_MOESM3_ESM.pdf]

Supplementary Table 3. Adjusted linear associations between sedentary time defined as a MET value of <1.75 and MVPA defined a MET value of ≥4.0 with subcomponents of metabolic risk and clustered metabolic risk in the *ADDITION-Plus* trial cohort (n=394)

	**Sedentary time (h/day), β (95% CI)**	**MVPA (h/day), β (95% CI)**
	**MET value: <1.75**	**MET value: ≥4.0**
**Waist (cm)**	0.74 (-0.03, 1.50)	-2.85 (-5.70, 0.01)
**Systolic blood pressure (mmHg)**	-0.46 (-1.45, 0.53)	-2.15 (-5.88, 1.58)
**HbA_{1c} (%)**	0.04 (-0.01, 0.10)	0.09 (-0.11, 0.29)
**Logged triacylglycerol (mmol/l (ln))**	0.02 (-0.01, 0.05)	0.05 (-0.07, 0.17)
**HDL-cholesterol (mmol/l)**	-0.01 (-0.03, 0.004)	-0.04 (-0.10, 0.02)
**zMS**	0.17 (0.02, 0.31) [*]	-0.04 (-0.59, 0.50)
**zMS** [a]	0.44 (0.06, 0.82) [*]	-0.02 (-0.30, 0.25)

Values for sedentary time and MVPA use individualised RMR [21] as definition of 1 MET.
All coefficients are adjusted for age, sex, intervention group, occupational socio-economic class, smoking status, sleep duration, total energy intake, percentage of energy from fat, and alcohol intake. Sedentary time is adjusted for MVPA and vice versa for MVPA. All outcomes except zMS and waist circumference are additionally adjusted for waist circumference. Systolic and diastolic blood pressure are additionally adjusted for use of anti-hypertensive drugs (yes/no); HbA_{1c} is additionally adjusted for use of glucose lowering drugs (yes/no); triacylglycerol is additionally adjusted for use of lipid-lowering drugs (yes/no); HDL-cholesterol is additionally adjusted for use of lipid-lowering drugs (yes/no); and zMS is additionally adjusted for use of anti-hypertensive drugs (yes/no), glucose lowering drugs (yes/no), and lipid-lowering drugs (yes/no).
zMS is a continuously distributed variable for clustered metabolic risk calculated by summing standardised values for waist circumference, triacylglycerol, HbA_{1c}, systolic blood pressure and the inverse of HDL-cholesterol.
[a] Difference in zMS per SD difference in sedentary time or MVPA
[*] $p<0.05$.
